# Systematic review and meta-analysis of enteral protein intake effects on growth in preterm infants

**DOI:** 10.1038/s41390-025-04115-9

**Published:** 2025-06-05

**Authors:** Maria Sanchez-Holgado, Mark J. Johnson, Ariadna Witte Castro, Susana Criado Camargo, Chris H. P. van den Akker, Patricia Alvarez-García, Marta Cabrera-Lafuente, Miguel Ángel Jiménez Varas, Miguel Saenz de Pipaon

**Affiliations:** 1 Department of Neonatology. La Paz University Hospital. Hospital La Paz Institute for Health Research-IdIPAZ, Madrid, Spain; 2 Faculty of Medicine, University of Southampton, Southampton, UK; 3 Department of Neonatal Medicine, University Hospital Southampton NHS Foundation Trust, Southampton, UK; 4 NIHR Biomedical Research Centre Southampton, University Hospital Southampton NHS Foundation Trust and University of Southampton, Southampton, UK; 5 Department of Pediatrics-Neonatology, Emma Children’s Hospital, Amsterdam UMC, University of Amsterdam, Amsterdam, The Netherlands; 6 Amsterdam Reproduction & Development Research Institute and Amsterdam Gastroenterology, Endocrinology & Metabolism Research Institute, Amsterdam, The Netherlands; 7 Information specialist. La Paz University Hospital, Madrid, Spain

## Abstract

**Background:**

The optimal enteral protein intake for adequate growth in preterm infants remains unclear. This systematic review evaluates the impact of protein intake from fortified human milk on growth in very preterm infants.

**Methods:**

Randomized clinical trials from January 2005 to August 2024 were included. Eligible studies measured true enteral protein intake in preterm infants. Searches were conducted in PubMed, Embase, and Cochrane CENTRAL. Risk of bias was evaluated using the revised Cochrane Risk of Bias Tool.

**Results:**

Ten randomized clinical trials (*n* = 646) were included. Meta-regression revealed a significant linear relationship between protein intake and weight gain (5.73 g/kg/day weight gain for each gram of protein/kg/day, *p* = 0.001), but not with head circumference or length gain. After adjustment for energy intake, significant relationships were found between protein intake and both weight gain and length growth. In contrast, the forest plot meta-analysis comparing high versus low protein intake showed no significant differences in weight or head circumference gain. However, infants receiving higher protein intake had greater weight at discharge (SMD 0.35, 95% CI 0.12 to 0.57, *n* = 312, 4 studies, high certainty) and more length growth (SMD 0.5, 95% CI 0.08 to 0.92, *n* = 174, 3 studies, moderate certainty).

**Discussion and Conclusion:**

Moderate to high-certainty evidence suggests that increased enteral protein intake improves growth outcomes in very preterm infants.

**REGISTRATION:**

PROSPERO CRD42022287991.

**Impact:**

- This systematic review is the first to evaluate the impact of enteral protein intake on growth in preterm infants <32 weeks, using studies that measured actual intake.
- A positive correlation was found between protein intake and weight gain. Meta-regression suggests most premature infants may require 4.0–4.5 g/kg/day to achieve in utero growth rates, rather than 3.5–4.0 g/kg/day.
- The meta-analysis indicates a positive relationship between protein intake, growth in length, and discharge weight.
- These findings underscore the critical role of adequate protein intake in growth outcomes and highlight the need to maintain appropriate energy: protein ratios.

## Introduction

Optimizing nutrition to achieve adequate growth and support neurodevelopmental outcomes is a critical challenge in preterm infants. Inadequate nutritional intake and poor growth during the first weeks of life are associated with poorer neurodevelopmental outcomes.^1–4^ Despite the emphasis on nutritional support for preterm infants, postnatal growth failure remains a significant issue with important prognostic implications for this vulnerable population.

Proteins are essential structural components of all human cells and play key roles in numerous physiologic processes, including functioning as enzymes, hormones, and transport proteins.^5^ Protein intake is considered the main driver of lean body mass acquisition, though it is also crucial to provide sufficient energy and micronutrients intake to support tissue growth.^5,6^ The optimal amount of protein and its effect on weight gain have been focal points of recent research.

The European Society for Pediatric Gastroenterology Hepatology and Nutrition (ESPGHAN) has recommended that the protein intake of preterm infants should be between and 3.5 and 4.0 g/kg/day, but may be increased up to 4.5 g/kg/day where growth is considered too slow.

However, these recommendations are based on information from a variety of sources and studies, some of which may not be robust. Therefore, the question of whether (even) more protein could be beneficial remains open.^5^ Reported protein intakes in clinical studies are often based on gross estimates of ingested milk volume and the estimated protein content of expressed milk, based on reference values or derived from occasional milk sample measurements. Such studies have shown improvements in anthropometric parameters, while others have reported no differences in weight gain, and it is likely that this is due to a limitation of this methodology.^7–12^ Consequently, the exact amount of daily protein required for adequate growth, and whether a protein intake higher than 4.0 g/kg/day increases growth, remains unclear.

Human milk is the first choice of milk for preterm infants, and it is associated with multiple short and long-term benefits.^13–17^ However, despite its advantages, human milk alone does not meet the nutritional requirements of preterm infants, particularly in terms of protein, energy, calcium, and phosphorus.^18,19^ Given the association between inadequate intake, especially of protein, and poor postnatal growth with adverse outcomes, human milk fortification with multicomponent fortifiers is considered essential.^14,20^

For preterm infants who do not achieve optimal growth with these fortifiers, protein intake can be further increased using modular protein supplements or by increasing the percentage of a multicomponent fortifier.^20–22^ Although various societies provide guidelines on enteral protein intake goals for fortified human milk in preterm infants,^5,23^ the evidence supporting these recommendations, as well as the necessity for mono- and multicomponent fortifiers, remains limited.

Therefore, we aimed to conduct a systematic review to assess the impact of protein intake on the growth of preterm infants, using data from studies where protein intakes were accurately measured.

## Methods

This systematic review was designed to evaluate the effect of enteral protein intake on growth in very preterm infants. Standard methods and Preferred Reporting Items for Systematic Reviews and Meta-Analyses (PRISMA) criteria were used.^24,25^ The protocol of this systematic review was registered at PROSPERO (CRD42022287991).

### Selection criteria

Randomized controlled trials (RCTs) published in English or Spanish between January 2005 and August 2024 that compared two different intakes of enteral protein in preterm infants with a gestational age <32 weeks were included, provided that true enteral protein intake was measured in one or both study groups. Additionally, only studies in which enteral protein intake was mainly provided as fortified human milk (>50% of enteral intake) were considered. Studies in which protein intake was not actually measured or those in which protein intake was predominantly provided as artificial formula were excluded. Studies in which the parenteral amino acid intakes were compared, were not included.

### Outcomes

The research questions were defined following the PICO framework^26,27^ (Table 1). The primary outcome was average weight gain in preterm infants during the intervention period in g/kg/day. Secondary outcomes were length growth (cm/week), head circumference growth (cm/week) and weight (g) at hospital discharge.

**Table 1:** Research question defined following the PICO framework.

PICO framework	
Population	Preterm newborns <32 weeks gestational age fed with fortified human milk
Intervention	Higher enteral protein intake
Comparison	Lower enteral protein intake
Outcome	Weight gain
	Length gain
	Head circumference gain
	Weight at hospital discharge

### Search strategy

PubMed, Embase and Cochrane CENTRAL (including ClinicalTrials.gov and ICTRP) were searched from January 2005 to August 2024 to identify eligible RCTs.

Searches were conducted by an information specialist (MAJV). The search strategy for each electronic database is given in Supplementary Material S1.

### Study selection

Titles and abstracts of the search results were independently reviewed by two reviewers (MSH and SCC) to select potential trials.

Trials unable to be retrieved were excluded. Papers that potentially answered the research question were reviewed in full text by the same two reviewers. Subsequently, these authors independently extracted data and cross-checked the results. Discrepancies were resolved by consensus in the research group.

### Data extraction

Reviewers MSH and SCC extracted the data independently using a standardized data collection form designed for the study.

The extracted data included a description of the studies and the comparison between the control and experimental groups. Each study was abstracted with the name of the first author, the reference, the country in which the study was conducted, the number of neonates included in each arm, gestational age, birth weight, protein intake, energy intake, method of protein intake measurement and frequency of measurement, duration of intervention, weight gain and other growth parameters.

### Risk of bias and certainty of evidence

The risk of bias was assessed for all outcomes analyzed from each included RCT using the revised Cochrane Risk of Bias Tool (RoB 2) for randomized trials.^28^ Elements evaluated were randomization process, deviations from the intended interventions, missing outcome data, measurement of the outcome and selection of the reported results. The risk of bias was assessed by two authors independently (MSH, SCC) and the results were subsequently cross-checked, and a conclusion was obtained.

Information about the magnitude of effect of the intervention, summary of data available and certainty of evidence for the meta-analysis results were presented in a ‘summary of findings’ table following the Grading of Recommendations, Assessment, Development and Evaluations (GRADE) guidelines.^29^ The certainty of evidence for each outcome was determined using the GRADEpro guideline development tool.^30^

### Data synthesis

To ascertain the relationship between protein intake (as measured) and growth (weight gain, length growth and head circumference growth) in a meta-analysis, mixed effects meta-regression was planned. This was chosen to account for the differing control and experimental protein intakes used across studies. This analysis included the RCTs where for at least one group (intervention or control) authors had conducted an actual measurement of protein intake, with this group then incorporated into the meta-regression. Only studies that provided sufficient data for the analysis (mean and SD, or sufficient data to allow these to be calculated) were included. This was carried out using the *metafor* package for R Studio 2024.09.0 Build 375 (Posit Software, PBC). Univariate meta-regression was planned to assess the relationship between protein intake and growth parameters, followed by a multivariate meta-regression to adjust for the differing energy intakes used across studies.

In addition, we planned to perform a conventional forest plot meta-analysis of studies providing data on protein intake, weight gain, length, and head circumference, and in which actual protein intake was measured in both the intervention and control groups.

Forest plots were created using Review Manager (RevMan) v5.4.1 (Cochrane Collaboration) using a random-effects model and inverse variance method. To summarize the results, given the continuous quantitative data, the Standardized Mean Difference (SMD) together with a 95% confidence interval (95% CI) was used as effect measure and a significance level of *P* < 0.05 was defined. Statistical heterogeneity was assessed using chi-squared (Chi^2^) test (*P* < 0.10 was considered significant) and the estimator *I*^2^, considering values under 25% low heterogeneity and values over 75% high heterogeneity.

In exceptional cases where weight gain was not reported in g/kg/day, but the study provided both the initial and final weights, as well as the duration of the intervention, weight gain velocity was calculated using the formula by Patel et al.^31^: [1000 x ln(Wn/W1)]/(Dn-D1), where W1 is the weight at the start and Wn is the weight at the final day of the observation. D1 is the starting day and Dn is the final day of the observation period (data estimated in this manner are indicated in Tables 2 and 3).

When the data was given in median (and interquartile ranges, IQRs) values, we used the quantile estimation (QE) method and the Box-Cox (BC) method of McGrath et al.^32–34^ as well as the Method for Unknown Non-Normal Distributions (MLN) approach of Cai et al.^35^ to estimate the sample mean and standard deviation.

For studies that only reported a mean but did not report standard deviation values, we imputed these values using the mean from other studies. This imputation was exclusively performed for the forest plot meta-analysis.

Sensitivity analyses were performed to evaluate the robustness of the results and the impact of the imputations. Possible causes of heterogeneity among study results were explored (subgroup analysis).

A funnel plot for publication bias was generated for the primary outcome.

## Results

### Study selection

A total of 3671 potential studies were found in PubMed, Cochrane central and Embase databases. After excluding duplicates, 2945 articles were assessed by title and/or abstract. We excluded 2885 as irrelevant. The full texts of 26 articles were assessed and reviewed for eligibility, resulting in 10 studies meeting the inclusion criteria.

**Fig. 1:**
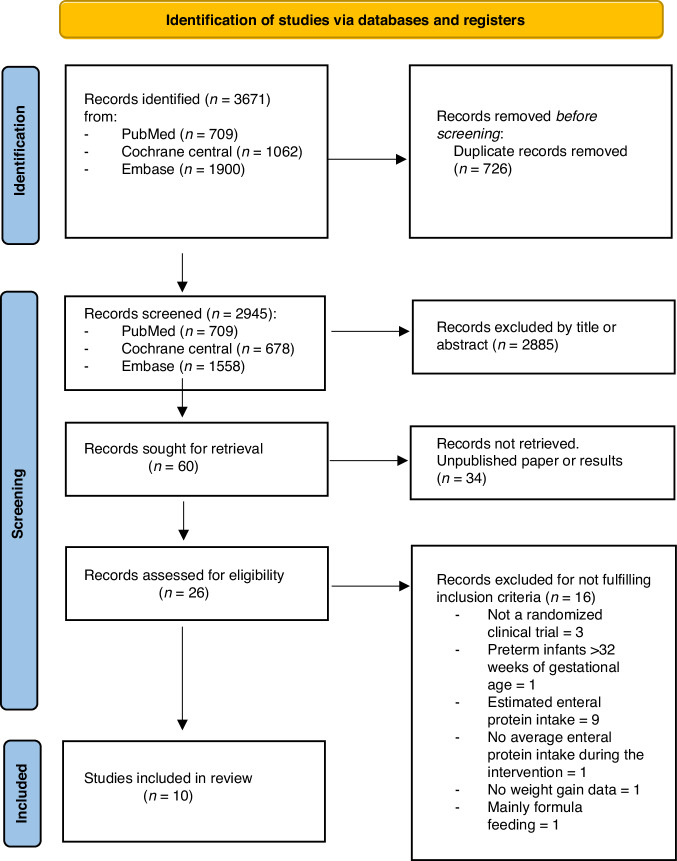
The Preferred Reporting Items for Systematic Review and Meta-Analyses flow chart of screening and selection of studies, with reasons for exclusion based on full-text review.

The PRISMA flowchart, for the study selection process, is shown in Fig. 1.

### Characteristics of included studies

A summary of characteristics of the individual studies can be found in Table 2 and Table 3. Overall, the ten included studies^36–45^ were relatively small, with sample sizes ranging between 32 and 120 participants and included a total of 646 preterm infants. The mean or median gestational age of participants ranged from 27 to 30 weeks, and their mean or median birth weights were between 968 and 1417 grams.

**Table 2:** Characteristics of included studies in the meta-analysis.

STUDY	POPULATION		INTERVENTION	ENTERAL INTAKES		HUMAN MILK MEASUREMENTS	GROWTH		RESULTS AND CONCLUSIONS
	Higher protein group	Lower protein group		Higher protein group	Lower protein group		Higher protein group	Lower protein group	
Maas^38^ Germany Single center trial	*n* = 30 GA (median, IQR) = 29.7 (27.9–31) weeks. BW (median, IQR) = 1193 (984–1326) g	*n* = 30 GA (median, IQR) = 30 (29–31.1) weeks. BW (median, IQR) = 1215 (1065–1393) g	Higher protein group: subdivided in two groups - standard fortification with multicomponent fortifier. -Targeted fortification based on HM analysis (on top of standard fortification). **Lower protein group:** standard fortification with multicomponent fortifier. **Enteral volume at the start of the intervention:** 100 ml/kg/day **Duration of intervention (**median, IQR) = 41 days (30–57) **Weight gain measurement time:** from birth to end of intervention	PROTEIN (mean, SD) 4.3 (0.27) g/kg/day **ENERGY** (mean, SD) 138 (6.4) kcal/kg/day	PROTEIN (mean, SD) 3.8 (0.24) g/kg/day ENERGY (mean, SD) 137 (9.2) kcal/kg/day	Frequency: twice a week Method: mid infrared Spectroscopy (MIRIS, Uppsala, Sweden)	WEIGHT GAIN Mean (SD) 16 (2.5) 16.3 (2.2) g/kg/day g/kg/day		An increase in protein intake by 0.6 g/kg/d to a mean intake of 4.3 g/kg/d did not enhance growth of very preterm infants. No differences in head circumference, length and lower leg longitudinal growth at discharge were detected.
							**LENGTH GROWTH** No reported data		
							**HEAD CIRCUMFERENCE GROWTH** No reported data		
							**WEIGHT AT HOSPITAL DISCHARGE** Mean (SD) 2669 (445)g 2611(420)g		
**Miller**^37^ **Australia** Multi-center trial	*n* = 43 GA (mean, SD) = 27.5 (2.2) wks. BW (mean, SD) = 1012 (315)g	*n* = 49 GA (mean, SD) = 28 (1.5) wks. BW (mean, SD) = 1056 (289) g	**Higher protein group:** standard fortification with multicomponent fortifier with higher protein fortifier (1.4 g protein/100 ml). **Lower protein group:** standard fortification with multicomponent fortifier with lower protein fortifier (1 g protein/100 ml) **Enteral volume at the start of the intervention:** 120 ml/kg/day **Duration of intervention** (mean, SD): - Higher protein group =74.3 (11.5) days - Lower protein group = 70.6 (9.2) days **Weight gain measurement time:** during intervention.	**PROTEIN** (median, IQR) 4.2 (3.6–4.7) g/kg/day **ENERGY** (median, IQR) 137 (119–149) kcal/kg/day	**PROTEIN** (median, IQR) 3.6 (3.2–4) g/kg/day **ENERGY** (median, IQR) 137 (122–150) kcal/kg/day	**Frequency:** once a week **Method:** infrared Spectroscopy (MilkoScan Minor, FOSS Analytical A/S, Hillerød, Denmark)	**WEIGHT GAIN** Median (IQR) 24 (20–28) 26 (24–28) g/day g/day		Infants in the higher-protein group achieved a greater weight at study end. There were no significant differences in lengths or head circumferences at study end. Fewer infants had lengths that were less than the 10th percentile at study end in the higher-protein group.
							**LENGTH GROWTH** Median (IQR) 1.15 (1.1–1.19) 1.09 (1.05–1.13) cm/wk cm/wk		
							**HEAD CIRCUMFERENCE GROWTH** Median (IQR) 0.94 (0.9–0.98) 0.95 (0.92–0.99) cm/wk cm/wk		
							**WEIGHT AT HOSPITAL DISCHARGE** Mean (SD) 2760 (498)g 2539 (494)g		
**McLeod**^43^ **Australia** Single center trial	*n* = 20 GA (mean, SD) = 27.1 (2) wks. BW (mean, SD) = 1009.2 (313.1)g	*n* = 20 GA (mean, SD) = 27 (1.9) wks. BW (mean, SD) = 1014.8 (269.3)g	**Higher protein group:** standard fortification with multicomponent fortifier **Lower protein group:** Targeted fortification based on HM analysis **Enteral volume at the start of the intervention:** 100 ml/kg/day **Duration of intervention (**mean, SD): - Higher protein group =42 (23) days - Lower protein group = 44 (24) days **Weight gain measurement time:** from birth to end of intervention	**PROTEIN** (mean, SD) 3.9 (0.3) g/kg/day **ENERGY** (mean, SD) 133.6 (8.12) kcal/kg/day	**PROTEIN** (mean, SD) 3.2 (0.4) g/kg/day **ENERGY** (mean, SD) 121.9 (9.32) kcal/kg/day	**Frequency:** once a week **Method:** mid infrared Spectroscopy (MIRIS, Uppsala, Sweden)	**WEIGHT GAIN** Mean (SD) 12.1 (1.6) 11.4 (1.4) g/kg/day g/kg/day		No differences were found in weight gain, weight, length and head circumference at discharge between the groups. Nutrition and growth was not improved by targeting milk fortification using measured milk composition compared with standard fortification.
							**LENGTH GROWTH** No reported data		
							**HEAD CIRCUMFERENCE GROWTH** No reported data		
							**WEIGHT AT HOSPITAL DISCHARGE** Mean (SD) 2464 (528)g 2294 (356)g		
**Rochow**^40^ **Canada** Single center trial	*n* = 52 GA (mean, SD) = 27.2 (1.2) wks. BW (mean, SD) = 980 (210) g	*n* = 51 GA (mean, SD) = 27.2 (1.7) wks. BW (mean, SD) = 980 (270) g.	**Higher protein group:** Targeted fortification based on HM analysis **Lower protein group:** standard fortification with multicomponent fortifier **Enteral volume at the start of the intervention:** 100 ml/kg/day **Duration of intervention** (mean, SD): - Higher protein group =27 (9) days - Lower protein group = 28 (10) days **Weight gain measurement time:** 21 days of intervention	**PROTEIN** (mean, SD) 4.5 (0.3) g/kg/day **ENERGY** (mean, SD) 140 (10) kcal/kg/day	**PROTEIN** (mean, SD) 3.6 (0.4) g/kg/day **ENERGY** (mean, SD) 121 (10) kcal/kg/day	**Frequency:** three times a week **Method:** Near-infrared milk analyzer (Unity SpectraStar, USA)	**WEIGHT GAIN** Mean (SD) 21.2 (2.5) 19.3 (2.4) g/kg/day g/kg/day		Protein intake was higher in targeted fortification group and was associated with greater weight gain. Infants in the intervention group from mothers with below-average breast milk protein content showed greatest impact on weight at 36 weeks, length, head circumference, fat, and fat-free mass.
							**LENGTH GROWTH** No reported data		
							**HEAD CIRCUMFERENCE GROWTH** No reported data		
							**WEIGHT AT HOSPITAL DISCHARGE** No reported data		
**Bulut**^39^ **Turkey** Single center trial	*n* = 16 GA (mean, SD) = 29.3 (1.9) wks. BW (mean, SD) = 1084 (219) g	*n* = 16 GA (mean, SD) = 29.3 (1.9) wks. BW (mean, SD) = 1143 (299) g	**Higher protein group:** Targeted fortification based on HM analysis **Lower protein group:** adjusted fortification based on BUN level. **Enteral volume at the start of the intervention:** 150 ml/kg/day **Duration of intervention**: 28 days **Weight gain measurement time:** during intervention.	**PROTEIN** (mean, SD) 4.5 (0.04) g/kg/day **ENERGY** (mean, SD) 145.02 (5.4) kcal/kg/day	**PROTEIN** (mean, SD) 4.01 (0.3) g/kg/day **ENERGY** (mean, SD) 144.3 (10.3) kcal/kg/day	**Frequency:** daily **Method:** mid infrared Spectroscopy (MIRIS, Uppsala, Sweden)	**WEIGHT GAIN** Mean (SD) 23.1 (4.3) 18.7 (4.3) g/kg/day g/kg/day		Targeted fortification had more positive effects on short-term growth compared with the adjustable fortification method (greater weight gain and higher weekly increase in head circumference). No significant differences in weekly increase in length were evident between the groups.
							**LENGTH GROWTH** Mean (SD) 1.04 (0.17) 0.93 (0.21) cm/wk cm/wk		
							**HEAD CIRCUMFERENCE GROWTH** Mean (SD) 0.98 (0.15) 0.84 (0.21) cm/wk cm/wk		
							**WEIGHT AT HOSPITAL DISCHARGE** No reported data		
**Khaira**^41^ **USA** Single center trial	n = 30 GA (mean, SD) = 27 (1.7) wks. BW (mean, SD) = 967.99 (245.4) g	n = 20 GA (mean, SD) = 27.3 (1.7) wks. BW (mean, SD) = 970.95 (217.9) g	**Higher protein group:** Targeted fortification based on HM analysis **Lower protein group:** standard fortification **Enteral volume at the start of the intervention:** 150 ml/kg/day **Duration of intervention**: variable, until 34–35 weeks’ gestation **Weight gain measurement time:** from 14 to 42 days of life	**PROTEIN** (mean) 4.5 g/kg/day	**PROTEIN** (mean) 4.24 g/kg/day	**Frequency:** every two weeks **Method:** Julie Z7 Automatic Milk Analyzer (Scope Electric Ltd, Germany)	**WEIGHT GAIN** Mean (SD) 16.8 (3.2) 17.1 (2.5) g/kg/day g/kg/day		No significant differences in growth velocity were observed between the groups.
							**LENGTH GROWTH** Mean (SD) 1.03 (0.4) 0.99 (0.47) cm/wk cm/wk		
							**HEAD CIRCUMFERENCE GROWTH** Mean (SD) 0.87 (0.5) 0.99 (0.8) cm/wk cm/wk		
							**WEIGHT AT HOSPITAL DISCHARGE** No reported data		
**Atchley**^36^ **USA** Single center trial	n = 16 GA (mean, SD) = 29.5 (1.6) wks. BW (mean, SD) = 1374.7 (282.8) g	n = 17 GA (mean, SD) = 30 (0.8) wks. BW (mean, SD) = 1416.9 (263.5) g	**Higher protein group:** enhanced protein diet (protein-energy ratio of 4 g/100 kcal) **Lower protein group:** standard protein diet (protein-energy ratio of 3 g/100 kcal) A targeted fortification method based on HM analysis was used in both groups. **Enteral volume at the start of the intervention:** 100 ml/kg/day **Duration of intervention**: 28 days. **Weight gain measurement time:** during intervention.	**PROTEIN** (mean, SD) 4.7 (0.3) g/kg/day **ENERGY** (mean, SD) 116.7 (5.8) kcal/kg/day	**PROTEIN** (mean, SD) 3.5 (0.3) g/kg/day **ENERGY** (mean, SD) 115 (6.35) kcal/kg/day	**Frequency:** once a week **Method:** information not provided.	**WEIGHT GAIN** Mean (95% CI) 21.6 (19.5–23.8) 19.1 (17–21.2) g/kg/day g/kg/day		No differences in weight gain or positive effects on the length and head circumference growth were observed between the groups. Preterm infants with a lower basal fat mass percentage who received a higher protein intake showed a more pronounced percentage fat accretion.
							**LENGTH GROWTH** Mean (95% CI) 1.26 (0.98–1.54) 1.4 (1.12–1.61) cm/wk cm/wk		
							**HEAD CIRCUMFERENCE GROWTH** Mean (95% CI) 0.98 (0.84–1.12) 1.19 (1.05–1.33) cm/wk cm/wk		
							**WEIGHT AT HOSPITAL DISCHARGE** No reported data		
**Gialeli**^42^ **Greece** Single center trial	n = 43 GA (mean, SD) = 29.7 (2.4) wks. BW (mean, SD) = 1182.9 (257.6) g	n = 77 GA (mean, SD) = 29.7 (2.5) wks. BW (mean, SD) = 1163.5 (221.3) g	**Higher protein group:** mother own milk was supplemented with preterm donor HM if required **Lower protein group:** mother own milk was supplemented with term donor HM if required A standard fortification was used in both groups when milk intake exceeded 50 ml/kg/day. **Enteral volume at the start of the intervention:** information not provided. Donor HM supplementation was only provided if mother’s own milk was not enough. **Duration of intervention**: 21 days **Weight gain measurement time:** during hospitalization	**PROTEIN** (mean, SD) 3.2 (0.6) g/kg/day **ENERGY** (median, IQR) 107 (96–116.5) kcal/kg/day	**PROTEIN** (mean, SD) 2.93 (0.54) g/kg/day **ENERGY** (median, IQR) 102.3 (92.6–111.2) kcal/kg/day	**Frequency:** once a week **Method:** mid infrared Spectroscopy (MIRIS, Uppsala, Sweden)	**WEIGHT GAIN** ^a^ Mean 13.32 g/kg/day 13.27 g/kg/day		The higher protein intake group had a better z-score trend for weight and head circumference from birth to the end of the donor milk period, and a higher mean body weight at discharge compared to the lower protein intake group.
							**LENGTH GROWTH** No reported data		
							**HEAD CIRCUMFERENCE GROWTH** No reported data		
							**WEIGHT AT HOSPITAL DISCHARGE** Mean (SD) 2560.7 (423.4)g 2411.8 (370.5)g		

Studies in which protein intake was measured in both the intervention and control group.

*GA* Gestational age, *BW* Birth weight, *HM* human milk, *BUN* blood urea nitrogen, *SD* Standard deviation, *IQR* interquartile range, *CI* confidence interval.

^a^Estimated using Patel’s formula,^31^ considering both birth weight and the weight at hospital discharge.

**Table 3:** Additional studies included in the meta-regression.

**Study**	**Population**	**Intervention**	**Enteral intakes**	**Human milk Measurements**	**Growth**	
**Kadioglu**^44^ **Turkey** Single center trial	**Higher protein groups:** - Adjusted fortification group: *n* = 20. GA (median, IQR) = 29 (28–31) wks. BW (median, IQR) = 1080 (905–1250) g. - Targeted fortification group: *n* = 20. GA (median, IQR) = 30 (27–31) wks. BW (median, IQR) = 980 (855–1105) g. Lower protein group: - Standard fortification group: *n* = 20. GA (median, IQR) = 29 (28–30) weeks. BW (median, IQR) = 1090 (880–1215) g.	**Higher protein groups:** - Adjusted fortification group based on BUN levels - Targeted fortification group based on HM analysis. **Lower protein group:** - Standard fortification group with multicomponent fortifier **Enteral volume at the start of the intervention:** 160 ml/kg/day **Duration of intervention**: 28 days **Weight gain measurement time:** during intervention	**Higher protein groups:** ***Adjusted fortification group*** PROTEIN: 4.3 g/kg/day ENERGY: 131 kcal/kg/day ***Targeted fortification group*** PROTEIN: 4.5 g/kg/day ENERGY: 133 kcal/kg/day **Lower protein group:** ***Standard fortification group*** PROTEIN: 3.6 g/kg/day ENERGY: 128 kcal/kg/day	**HM analysis was conducted exclusively in targeted fortification group**. **Frequency:** twice a week **Method:** mid infrared Spectroscopy (MIRIS, Uppsala, Sweden)	**Higher protein groups:** ***Adjusted fortification group*** WEIGHT GAIN Median (IQR) 23.5 (22–26) g/kg/day LENGTH GROWTH Median (IQR) 1.4 (1.05–1.4) cm/week HEAD CIRCUMFERENCE GROWTH Median (IQR) 0.88 (0.7–1.05) cm/week ***Targeted fortification group*** WEIGHT GAIN Median (IQR) 25.5 (21–28) g/kg/day LENGTH GROWTH Median (IQR) 1.23 (1.05–1.32) cm/week HEAD CIRCUMFERENCE GROWTH Median (IQR) 0.7 (0.7–0.88) cm/week	**Lower protein group:** ***Standard fortification group*** WEIGHT GAIN Median (IQR) 12 (9–17) g/kg/day LENGTH GROWTH Median (IQR) 0.7 (0.53–0.8) cm/week HEAD CIRCUMFERENCE GROWTH Median (IQR) 0.61 (0.35–0.7) cm/week
**Olhager**^45^ **Sweden** Single center trial	**Higher protein groups:** *n* = 29. GA (mean SD) = 28.3 (1.6) weeks. BW (mean, SD) = 1079 (297) g. **Lower protein group:** *n* = 27. GA (mean SD) = 29 (1.8) weeks. BW (mean, SD) = 1148 (305) g.	**Higher protein group:** Standard fortification with multicomponent fortifier **Lower protein group:** Targeted fortification based on HM analysis **Enteral volume at the start of the intervention:** No information is available **Duration of intervention**: until 36 weeks gestational age. **Weight gain measurement time:** until 40 weeks gestational age	**Higher protein group:** PROTEIN: 4.2 (0.6) g/kg/day ENERGY: 161 (14) kcal/kg/day **Lower protein group:** PROTEIN: 3.6 (0.4) g/kg/day ENERGY: 150 (16) kcal/kg/day	**HM analysis was conducted exclusively in targeted fortification group**. **Frequency:** once a week **Method:** mid infrared Spectroscopy (MIRIS, Uppsala, Sweden)	**Higher protein group:** WEIGHT GAIN ^a^ Mean 12.96 g/kg/day LENGTH GROWTH ^b^ Mean(SD) 0.8 (0.4) cm/week HEAD CIRCUMFERENCE GROWTH ^b^ Mean (SD) 1.13 (1) cm/week	**Lower protein group:** WEIGHT GAIN ^a^ Mean 12.72 g/kg/day LENGTH GROWTH ^b^ Mean (SD) 0.71 (0.5) cm/week HEAD CIRCUMFERENCE GROWTH ^b^ Mean (SD) 0.85 (0.3) cm/week

Studies in which at least one group (intervention or control) performed an actual measurement of protein intake.

*GA* Gestational age, *BW* Birth weight, *HM* human milk, *BUN* blood urea nitrogen, *SD* Standard deviation, *IQR* interquartile range, *CI* confidence interval.

^a^Estimated using Patel’s formula,^31^ considering both birth weight and the weight at 40 weeks of gestational age.

^b^From birth to 28 days of life.

The trials were conducted in Europe,^38,42,45^ Turkey,^39,44^ Australia,^37,43^ Canada^40^ and United States of America.^36,41^ Four studies showed positive effects of higher protein intake (enteral protein intake range 3.2**–**4.5 g/kg/d) vs lower protein intake (2.9**–**4.0 g/kg/d) on weight gain,^39,40,42,44^ whereas six studies were not able to detect differences on weight gain between higher (3.9**–**4.7 g/kg/d) vs lower protein intake (3.2–4.2 g/kg/d).^36–38,41,43,45^

All included studies compared methods of human milk fortification, resulting in different enteral protein intakes, and all measured the nutrient content of mother’s milk in one or both study groups.

Five studies^38,40,41,43,45^ compared *standard fortification method*, which involves adding a fixed amount of multicomponent fortifier throughout the entire fortification period,^18,20,22^ with a *targeted fortification method* that tailors fortification to the actual macronutrient composition of human milk.^46,47^ One study^39^ compared targeted fortification with *adjusted fortification*,^48–51^, which uses blood urea nitrogen (BUN) concentration as a surrogate marker for protein intake. On the other hand, Kadıoglu et al.^44^ compared the three methods: standard fortification, adjusted fortification, and targeted fortification.

Atchley et al.^36^ conducted a comparative study of targeted fortification with different target enteral protein intakes between the groups.

The supplements added to human milk in targeted fortification varied across studies.

Miller et al.^37^ evaluated standardized fortification with multicomponent fortifiers containing varying amounts of protein across groups. Gialeli et al.^42^ conducted standardized fortification but the human milk was supplemented, if necessary, with donated human milk with different protein content (preterm vs. term donated human milk).

### Risk of bias assessment

**Fig. 2:**
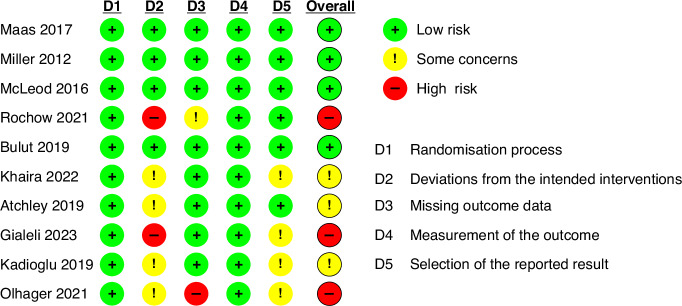
Risk of bias assessment of included studies.

The risk of bias of included studies is presented in Fig. 2. Four of the included studies had a low risk of bias.^37–39,43^ Three studies had some concerns^36,41,44^ primarily due to the absence of a pre-specified analysis plan in a registered protocol. Three studies were considered to have a high risk of bias,^40,42,45^, largely due to the lack of an adequate intention-to-treat analysis, potentially affecting the validity of the treatment effect estimation. Overall, the risk of bias assessment highlights some variability in the methodological quality of the included studies.

### Primary outcome- Meta-regression of protein intake vs growth

**Fig. 3:**
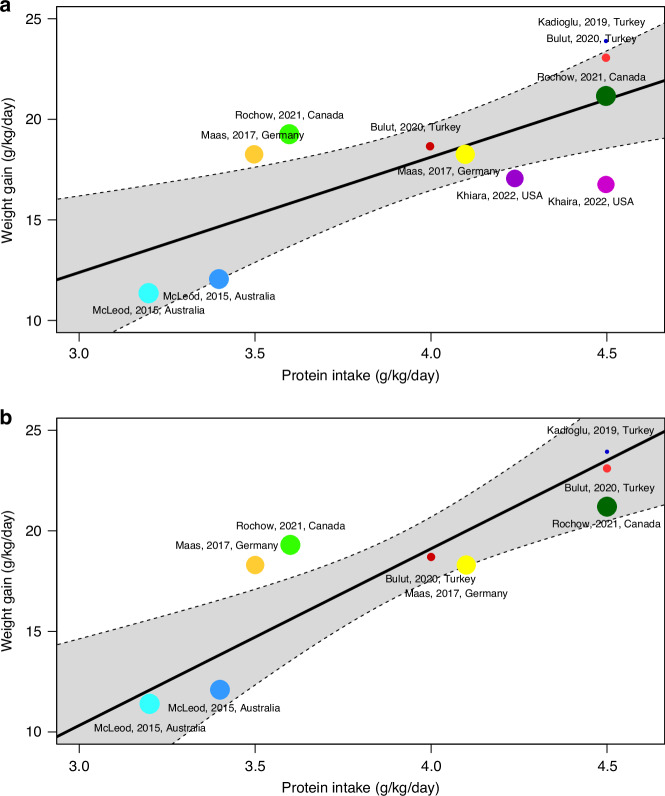
Meta-regression bubble plots for weight gain in relation to measured protein intake. The size of each bubble represents the weight of each study in the analysis, the black line is the line of regression and the shaded area within the dashed line is the 95% confidence interval of the regression line. **a** Unadjusted analysis showing a significant linear relationship between protein intake and weight gain with 5.73 g/kg/day weight gain for each g/kg/day protein, *p* ≤ 0.001. **b** Analysis after adjustment for concurrent energy intakes showing a significant linear relationship between protein intake and weight gain with 8.78 g/kg/day weight gain for each g/kg/day protein, *p* ≤ 0.001.

For the analysis of protein intake in relation to weight gain, the studies by Bulut et al.^39^ (both intervention and control groups), Maas et al.^38^ (both intervention and control groups), Rochow et al.^40^ (both intervention and control groups), McLeod et al.^43^ (both intervention and control groups), Kadioglu et al.^44^ (targeted fortification group only) and Khaira et al.^41^ (both intervention and control groups) were included. We observed a significant linear relationship between protein intake and weight gain (Fig. 3a), with each additional gram per kg per day of protein intake resulting in an increase of 5.73 g/kg/day in weight gain (Table 4). After adjusting for the energy intake in the included studies, this effect size increased to 8.78 g/kg/day for each additional gram per kg per day of protein intake (Fig. 3b and Table 4).

**Table 4:** Results of meta-regression analysis showing effect of protein intake on weight gain, head circumference gain and length gain, both unadjusted and after adjustment for concurrent energy intake.

	Weight Gain (g/kg/day)		Head Circumference gain (mm/week)		Length gain (mm/week)	
Meta-regression	Univariate effect size (95% CI)	Multivariate effect size (95% CI)	Univariate effect size (95% CI)	Multivariate effect size (95% CI)	Univariate effect size (95% CI)	Multivariate effect size (95% CI)
Protein intake (g/kg/day)	5.73* (2.27 to 9.20)	8.78* (4.38 to 13.19)	−0.64 (−2.67 to 1.38)	−0.72 (−2.95 to 1.51)	0.37 (−2.62 to 3.35)	0.78* (0.35 to 1.21)
Energy Intake (kcal/kg/day)	N/A	−0.13 (−0.39 to 0.14)	N/A	0.09 (−0.03 to 0.22)	N/A	−0.26* (−0.20 to −0.10)

*p ≤ 0.001.

Statistically signifcant results have been highlighted using bold.

For the analysis of protein intake in relation to both head circumference and length gain, the studies by Bulut et al.^39^ (both intervention and control groups), Kadioglu et al.^44^ (targeted fortification group only), Khaira et al.^41^ (both intervention and control groups), Olhager et al.^45^(individual fortification group only) and Miller et al.^37^ (both intervention and control groups) were included. There was no effect of protein intake on length or head circumference gain in the unadjusted meta-regression model (Table 4). However, the relationship between protein intake and length gain became significant once energy intake was adjusted for, with each gram per kg per day of protein intake associated with a 0.78 mm per week increase in length (Table 4 and Supplementary Material S2a). Interestingly, in this adjusted analysis, energy intake had a negative influence on length gain, with each additional kcal per kg day of energy intake associated with a 0.26 mm per week reduction in length gain (Table 4 and Supplementary Material S2b).

### Secondary outcome—Forest plot meta-analysis of protein intake vs growth

**Fig. 4:**
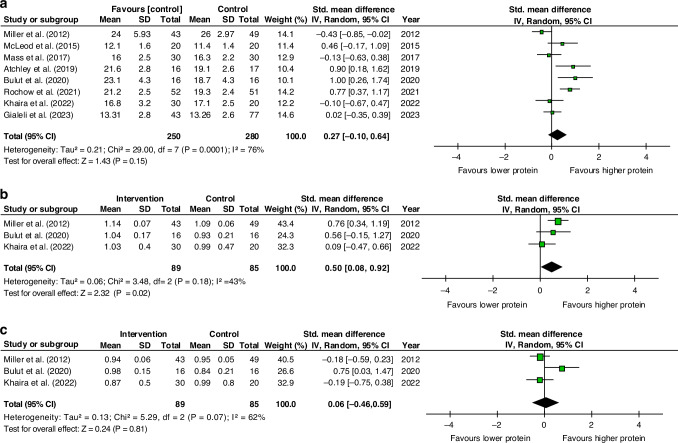
Forest plot meta-analysis of the effect of enteral protein intake on growth outcomes in preterm infants. **a** Weight gain for higher vs lower enteral protein intake, showing no significant difference. **b**Length gain, for higher vs lower enteral protein intake, showing a mean increase in length gain of 0.5 cm/week for higher protein intakes, *p* = 0.02. **c** Head circumference gain for higher vs lower enteral protein intake, showing no significant difference.

Eight studies had data available for the forest plot meta-analysis^36–43^ (Fig. 4). We did not find any effect in weight gain between the groups (Fig. 4a). The heterogeneity was considered high.

We performed a sensitivity analysis, removing Gialeli et al.^42^ and Atchley et al.^36^ as these studies had not presented the standard deviation (SD), meaning this had needed to be imputed. The result remained non-significant (SMD = 0.24, 95%CI [−0.23, 0.71], *P* = 0.33) and the heterogeneity was considered high (*I*² = 80%; *P* < 0.0002). A sensitivity analysis was also performed, excluding the two studies with high risk of bias^40,42^ and the results remained non-significant (S*MD* = 0.23, 95%CI [−0.23, 0.69], *P* = 0.33).

For the evaluation of the other secondary outcomes, only studies that provided the necessary data on length growth^37,39,41^ and head circumference growth^37,39,41^ were included in the meta-analysis. Length gain was significantly greater in the group receiving additional protein (Fig. 4b), but head circumference gain in cm/week was similar between groups (Fig. 4c).

No publication bias was observed in the visual inspection of the funnel plot (Supplementary Material S3). However, its value is limited by the small number of included studies.

### Subgroup analysis

Due to the detected heterogeneity and the overlap in the compared protein intake ranges, a subgroup analysis was conducted, including only those studies in which the higher protein intake group received more than 4.0 g/kg/day.^36–41^ This analysis did not find a significant relationship between a protein intake greater than 4.0 g/kg/day and weight gain (SMD = 0.30, 95% CI [−0.21, 0.81], *P* = 0.24, see Supplementary Material S4).

### Secondary outcomes—Forest plot meta-analysis of protein intake vs weight at discharge

**Fig. 5:**
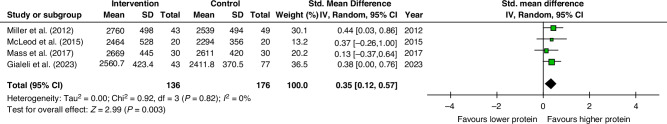
Forest plot meta-analysis of discharge weight in preterm infants comparing higher versus lower enteral protein intake.

For the evaluation of weight gain at hospital discharge, only studies that provided the necessary data were included.^37,38,42,43^ Weight at discharge was significantly greater in the group receiving higher protein intake (Fig. 5).

### Summary of findings. Certainty of evidence

The certainty of evidence, according to the GRADE guidelines, was graded low for the weight gain due to heterogeneity and imprecision. The evidence was downgraded for other outcomes for small sample size or inconsistency (Table 5).

**Table 5:** Summary of findings (certainty of evidence*)*.

Certainty assessment							№ of patients		Effect		Certainty	Importance
№ studies	Study design	Risk of bias	Inconsistency	Indirectness	Imprecision	Other considerations	Higher protein intake	Lower protein intake	Relative (95% CI)	Absolute (95% CI)		
**Weight gain (g/kg/day)**												
8	Randomized trials	Not serious	Serious^a^	Not serious	Serious^b^	None	250	280	—	SMD **0.27 SD higher** (-0.1 lower to 0.64 higher)	⨁⨁◯◯ Low	IMPORTANT
**Length gain (cm/week)**												
3	Randomized trials	Not serious	Not serious	Not serious	Serious^c^	None	89	85	—	SMD **0.5 SD higher** (0.08 higher to 0.92 higher)	⨁⨁⨁◯ Moderate	IMPORTANT
**Head circumference gain (cm/week)**												
3	Randomized trials	Not serious	Serious^a^	Not serious	Serious^b^	None	89	85	—	SMD **0.06 SD higher** (−0.46 lower to 0.59 higher)	⨁⨁◯◯ Low	IMPORTANT
**Weight at discharge (grams)**												
4	Randomized trials	Not serious	Not serious	Not serious	Not serious	None	136	176	—	SMD **0.35** **SD higher** (0.12 higher to 0.57 higher)	⨁⨁⨁⨁ High	IMPORTANT

*CI* confidence interval, *SMD* standardized mean difference.

^a^High heterogeneity in included studies.

^b^The confidence interval is considered imprecise because it includes the null value.

^c^Small sample size.

## Discussion

This systematic review and meta-analysis assessing the association between enteral protein intake and growth in preterm infants with less than 32 weeks of gestation demonstrated a significant linear relationship between protein intake and weight gain. Our review is unique in that it includes only clinical trials with accurate measurements of actual protein intake and comprehensive analyses of growth throughout the enteral nutrition phase, coupled with a focus on studies in which enteral protein intake was provided as fortified human milk.^52,53^

Protein is a critical nutrient for preterm infants to achieve an intrauterine-like growth rate.^5,54^ Using meta-regression, we found that aiming for growth rates of 20 g/kg/day—similar to those of a fetus at 23–25 weeks^5^—would require approximately 4.4 g/kg/day of protein intake. Similarly, achieving growth rates of 17 g/kg/day, comparable to a fetus at around 28 weeks, would require 4 g/kg/day. This suggests the most preterm infants may require upward of the 4 g/kg/day recommended as standard by ESPGHAN,^5^ and perhaps need closer to the maximum recommended amount of 4.5 g/kg/day in order to achieve in utero growth rates. However, this conclusion should be interpreted with caution for infants at the extremes of prematurity, given that the median gestational ages of the infants included in our analyses were 27–30 weeks.

When adjusting for concurrent energy intakes, the impact of protein intake on weight gain was more apparent, with meta regression suggesting that protein intakes of 3.8–4.2 g/kg/d are needed to achieve growth rates of 17–20 g/kg/d, more in keeping with current standard recommendations by ESPGHAN.^5^

Meta-regression showed no significant linear relationship between protein intake and head circumference and length gain, but there was an effect seen on length gain after adjusting for energy intake. This revealed a positive impact of protein intake on length, but interestingly, showed a negative association between length gain and energy intake when adjusting for protein. This suggests better length gain is seen in intakes of 130–140 kcal/kg/day, at the upper end of the recent standard EPSGHAN intake recommendation.^5^ This finding is in keeping with the concept of maintaining energy: protein ratios, suggesting that the right balance of energy and protein is needed to achieve linear growth, with excessive energy resulting in fat deposition and weight gain rather than linear growth. Moreover, micronutrients are critical for supporting growth, particularly potassium, phosphate, essential fatty acids, and a variety of vitamins and trace elements.^55^

In contrast to the meta-regression, the forest plot meta-analysis did not reveal a significant difference in the rate of weight gain between studies comparing low-dose versus high-dose protein. Nonetheless, there were differences between these groups in terms of both length growth and discharge weight in favor of the higher protein intake group.

This discrepancy may be due to several factors, such as variability in the protein intake levels considered “high” across the different studies, with overlap between intervention groups in one study and control groups in another, as well as the heterogeneity observed in the results. This overlap may hinder the ability to draw clear conclusions regarding the effects of different protein intake levels, particularly considering that the recommended intake falls within this range. To address the issue of overlap in our study on protein intake, we employed meta-regression analysis.

In this context, meta-regression provides a more detailed assessment of the quantitative relationship between actual protein intake and growth rates compared to traditional meta-analyses. In addition, meta-regression allowed for adjustment of concurrent energy intakes, a lack of which has been a criticism of previous similar meta-analyses in this area. Furthermore, the studies varied in their growth velocity measurement periods and protein fortification methods, which may have affected the quality of the evidence in this analysis. These limitations underscore the need for further clinical trials with well-defined enteral protein intake levels to accurately assess the impact of protein intake on growth in this population. Nevertheless, despite these challenges, our findings from the meta-regression analysis reveal a positive correlation between higher enteral protein intake and enhanced weight gain.

Three systematic reviews on enteral protein intake and growth in preterm infants have been published previously. Tonkin et al.^7^ conducted a systematic review comparing different levels of enteral protein intake with various feeding methods (formula, unfortified human milk, and human milk fortified with different protein levels). Inclusion criteria also included RCT study designs and premature infants as the study population. Of the seven studies that evaluated growth with fortified human milk, four found a positive effect of increased protein intake on growth. However, a quantitative meta-analysis of the results could not be performed, limiting the ability to draw statistically significant conclusions. Additionally, only three studies reported actual protein intake. Given the variability of the composition of human milk, assuming an amount of protein may lead to significant variations from actual protein intake received by the infant, limiting the establishment of accurate conclusions.^56^

Fenton et al.^57^ recently conducted a systematic review and meta-analysis to evaluate the impact of higher protein intake on the growth of very low birth weight infants. Only two clinical trials were included in the final meta-analysis. Although these studies maintained constant energy intake while increasing protein intake, facilitating the analysis of the exclusive effect of enteral protein intake, several limitations were noted. Both studies analyzed preterm infants fed exclusively with artificial formula, and the trials, published in 1991 and 1994, compared protein intakes between 2.6–3.8 g/kg/day and 2.8–3.6 g/kg/day, respectively. These findings have limited applicability to current neonatal care, where preterm infants are predominantly fed fortified human milk, and the primary question focuses on whether protein intakes exceeding 4 g/kg/day can further improve growth. Wang et al.^58^ included three observational studies to assess the relationship between nutrient intake and growth in preterm infants during the transition phase from parenteral to enteral nutrition, thereby analyzing both enteral and parenteral protein intake.

The main strength of this study is the exclusive inclusion of studies in which actual protein intake was measured through regular analysis of human milk. Limitations include the low number of studies included, the heterogeneity detected, and the need to estimate some of the data provided by the studies to perform the meta-analysis. Also, we solely selected studies where actual protein content in milk was truly measured. Thus, RCTs comparing high versus low protein intake, but where actual milk protein content was not measured, were excluded. This may be considered as a selection bias with regard to the forest plot meta-analysis. Additionally, the search timeframe was restricted to the past 20 years to ensure the systematic review incorporates findings based on accurate and reliable protein intake measurements, made possible by the availability of human milk composition analyzers.^59^ Another limitation of our review is that it did not evaluate the long-term outcomes of enteral protein intake in this population, such as neurodevelopmental outcomes or long-term growth. These aspects are crucial for understanding the full impact of early nutritional interventions and warrant further investigation in future research.

In conclusion, our study provides evidence of the effect of protein intake on growth, representing the first systematic review to include studies measuring actual protein intake in preterm infants younger than 32 weeks. It also suggests the most premature infants may need intakes around 4.0 or perhaps even 4.5 g/kg/day to achieve in utero growth rates, rather than the lower limit of the ESPGHAN recommendation of 3.5 g/kg/day.^5^ It also confirms that, after adjusting for energy intake, protein intake has a positive impact on linear growth. Higher length growth rates may occur closer to the upper end of the ESPGHAN standard recommended energy intakes of 130–140 kcal/kg/day, highlighting the importance of ensuring adequate protein intake and energy: protein ratios to improve outcomes in this vulnerable population.

## Supplementary information

Supplementary material S1

PRISMA 2020 Main Checklist

## Data Availability

The datasets generated during and/or analyzed during the current study are available from the corresponding author on reasonable request. Researchers submitting a methodologically sound proposal should contact M.Johnson@soton.ac.uk. In order to access, requesters will need to sign a data access agreement.
