# Supplementary material for: Systematic review and meta-analysis of enteral protein intake effects on growth in preterm infants
Source: Pediatr Res. 2025 Jun 5;98(5):1696–710. doi: 10.1038/s41390-025-04115-9 (PMC12602309; doi:10.1038/s41390-025-04115-9)
Supplement: Supplementary file 1 — Supplementary material S1 [file 41390_2025_4115_MOESM1_ESM.pdf]

Supplementary material S1.Full search strategies

Pubmed:
("Proteins"[MeSH Terms] OR "protei*"[Title/Abstract]) AND ("Milk, Human"[Mesh] OR milk*[tiab] OR "Enteral Nutrition"[Mesh] OR Enteral[Title/Abstract] OR Feeding[Title/Abstract] OR DHM[tiab] OR HM[tiab] OR breastmilk*[tiab] OR "Infant Formula"[MeSH Terms] OR "infant formula*"[Title/Abstract] OR "baby formula*"[Title/Abstract] OR ("formula*"[Title/Abstract] AND ("Baby"[Title/Abstract] OR "Babies"[Title/Abstract] OR "infant*"[Title/Abstract]))) AND ("Child Development"[MeSH Terms] OR "Growth"[MeSH Terms] OR "Body Size"[MeSH Terms] OR "growth*"[Title/Abstract] OR "weight*"[Title/Abstract] OR "lenght*"[Title/Abstract] OR "infant development"[Title/Abstract] OR "Child Development"[Title/Abstract] OR "head circumference"[Title/Abstract]) AND ("infant"[MeSH Terms] OR "infant*"[Title/Abstract] OR "prematur*"[Title/Abstract] OR "preterm"[Title/Abstract] OR "pre term"[Title/Abstract] OR "low birth weight"[Title/Abstract] OR "low birthweight"[Title/Abstract] OR "VLBW"[Title/Abstract] OR "LBW"[Title/Abstract] OR "micropremie"[Title/Abstract] OR "toddler*"[Title/Abstract]) AND ("Randomized Controlled Trial"[Publication Type] OR "Controlled Clinical Trial"[Publication Type] OR "Randomized Controlled Trial"[Title/Abstract] OR "randomized clinical trial"[Title/Abstract] OR "RCT"[Title/Abstract] OR "clinical trial"[Title/Abstract] OR "clinical trial"[Publication Type] OR "clinical trials as topic"[MeSH Terms] OR "double-blind method"[MeSH Terms] OR (randomized[Title/Abstract] AND trial*[Title/Abstract]) OR ((single[Title/Abstract] OR double*[Title/Abstract] OR triple*[Title/Abstract] OR treble[Title/Abstract]) AND (blind*[Title/Abstract] OR mask*[Title/Abstract]))) AND ("2005/01/01"[Date - Publication] : "2024/08/01"[Date - Publication])

Central
#1 MeSH descriptor: [Proteins] explode all trees 127425
#2 protei*:ti,ab 68261
#3 #1 OR #2 175874
#4 MeSH descriptor: [Milk, Human] explode all trees 1099
#5 (milk* OR enteral OR feeding OR DHM OR HM OR breastmilk* OR 'infant formula*' OR 'baby formula*'):ti,ab 30001
#6 MeSH descriptor: [Enteral Nutrition] explode all trees 1965
#7 MeSH descriptor: [Infant Formula] explode all trees 676
#8 formula*:ti,ab 44881
#9 (baby OR babies OR infant*):ti,ab 47791
#10 #8 AND #9 4133
#11 #4 OR #5 OR #6 OR #7 OR #10 31183
#12 MeSH descriptor: [Child Development] explode all trees 2732
#13 MeSH descriptor: [Growth] explode all trees 21306
#14 MeSH descriptor: [Body Size] explode all trees 25441

#15 ('Child Development' OR growth* OR weight* OR lenght* OR 'infant Development' OR 'head circumference'):ti,ab 162762
#16 #12 OR #13 OR #14 OR #15 172481
#17 MeSH descriptor: [Infant] explode all trees 34222
#18 (infant* OR prematur* OR preterm OR 'pre term' OR 'low birth weight' OR 'low birthweight' OR VLBW OR LBW OR micropremie OR toddler*):ti,ab 74180
#19 #17 OR #18 91075
#20 #3 AND #11 AND #16 AND #19 **1398**

EMBASE

(protei*:ab,ti OR 'protein'/exp) AND ('breast milk'/exp OR 'enteric feeding'/exp OR 'artificial milk'/exp OR milk*:ab,ti OR enteral:ab,ti OR feeding:ab,ti OR dhm:ab,ti OR hm:ab,ti OR breastmilk*:ab,ti OR 'infant formula*':ab,ti OR 'baby formula*':ab,ti) AND ('postnatal development'/exp OR 'child development and growth'/exp OR 'postnatal growth'/exp OR 'body size'/exp OR 'length'/exp OR 'weight'/exp OR 'head circumference'/exp OR 'child development':ab,ti OR growth*:ab,ti OR weight*:ab,ti OR lenght*:ab,ti OR 'infant development':ab,ti OR 'head circumference':ab,ti) AND ('prematurity'/exp OR infant*:ab,ti OR prematur*:ab,ti OR preterm:ab,ti OR 'pre term':ab,ti OR 'low birth weight':ab,ti OR 'low birthweight':ab,ti OR vlbw:ab,ti OR lbw:ab,ti OR micropremie:ab,ti OR toddler*:ab,ti OR 'infant'/exp) AND [2005-2023]/py AND ('randomized controlled trial'/exp OR 'randomized controlled trial':ab,ti OR 'randomized clinical trial':ab,ti OR rct:ab,ti OR 'controlled clinical trial'/exp OR 'clinical trial'/de OR 'controlled clinical trial'/de OR 'controlled study'/de OR 'double blind procedure'/de OR 'randomized controlled trial'/de) AND [01-01-2005]/sd NOT [01-08-2024]/sd

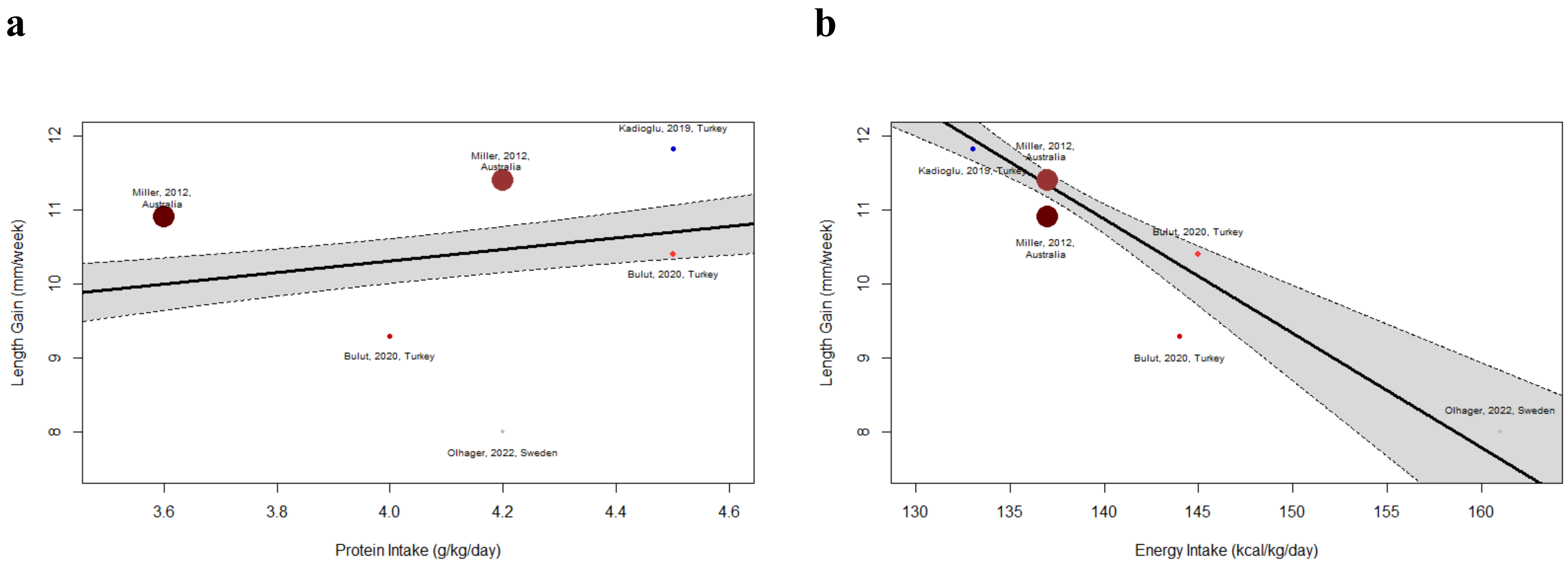

Figure S2. Meta regression bubble plots for length gain in relation to measured protein intake after adjustment for energy intake in each study (a) and length gain in relation to measured energy intake after adjustment for protein intake in each study (b). The size of each bubble represents the weight of each study in the analysis, the black line in the line of regression and the shaded area within the dashed line is the 95% confidence interval of the regression line.

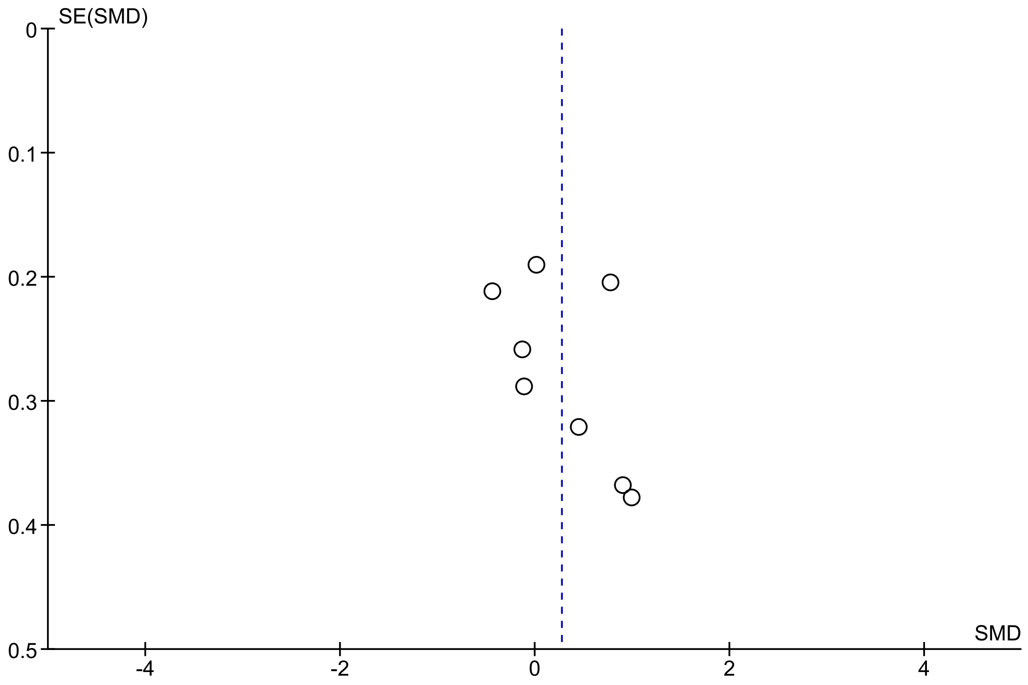

Figure S3. Funnel plot. Studies included in the main outcome meta-analysis.

Study or Subgroup	Experimental Mean	Experimental SD	Experimental Total	Control Mean	Control SD	Control Total	Weight	Std. Mean Difference IV, Random, 95% CI	Year
**1.6.1 >4 g/kg/day**									
Miller et al. 2012	24	5.93	43	26	2.97	49	14.1%	-0.43 [-0.85, -0.02]	2012
Mass et al. 2017	16	2.5	30	16.3	2.2	30	12.9%	-0.13 [-0.63, 0.38]	2017
Atchley et al. 2019	21.6	2.8	16	19.1	2.6	17	10.4%	0.90 [0.18, 1.62]	2019
Bulut et al. 2020	23.1	4.3	16	18.7	4.3	16	10.1%	1.00 [0.26, 1.74]	2020
Rochow et al. 2021	21.2	2.5	52	19.3	2.4	51	14.2%	0.77 [0.37, 1.17]	2021
Khaira et al. 2022	16.8	3.2	30	17.1	2.5	20	12.2%	-0.10 [-0.67, 0.47]	2022
**Subtotal (95% CI)**			**187**			**183**	**74.0%**	**0.30 [-0.21, 0.81]**	
Heterogeneity: Tau² = 0.32; Chi² = 27.42, df = 5 (P < 0.0001); I² = 82%									
Test for overall effect: Z = 1.17 (P = 0.24)									
**1.6.2 <4 g/kg/day**									
McLeod et al. 2015	12.1	1.6	20	11.4	1.4	20	11.4%	0.46 [-0.17, 1.09]	2015
Gialeli et al. 2023	13.31	2.8	43	13.26	2.6	77	14.6%	0.02 [-0.35, 0.39]	2023
**Subtotal (95% CI)**			**63**			**97**	**26.0%**	**0.16 [-0.24, 0.56]**	
Heterogeneity: Tau² = 0.03; Chi² = 1.38, df = 1 (P = 0.24); I² = 27%									
Test for overall effect: Z = 0.79 (P = 0.43)									
**Total (95% CI)**			**250**			**280**	**100.0%**	**0.27 [-0.10, 0.64]**	
Heterogeneity: Tau² = 0.21; Chi² = 29.00, df = 7 (P = 0.0001); I² = 76%									
Test for overall effect: Z = 1.43 (P = 0.15)									
Test for subgroup differences: Chi² = 0.18, df = 1 (P = 0.67), I² = 0%									

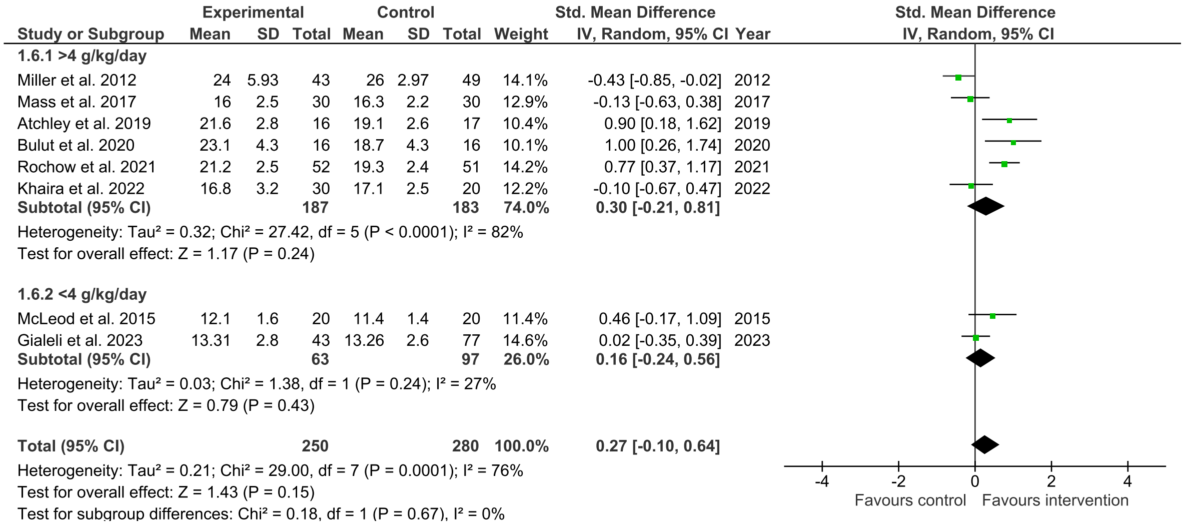

Figure S4. Subgroup analysis
